# Enhancing Understanding of the Visual Cycle by Applying CRISPR/Cas9 Gene Editing in Zebrafish

**DOI:** 10.3389/fcell.2018.00037

**Published:** 2018-04-11

**Authors:** Rebecca Ward, Husvinee Sundaramurthi, Valeria Di Giacomo, Breandán N. Kennedy

**Affiliations:** ^1^UCD School of Biomolecular & Biomedical Science, UCD Conway Institute, University College Dublin, Dublin, Ireland; ^2^UCD School of Medicine, University College Dublin, Dublin, Ireland; ^3^Systems Biology Ireland, University College Dublin, Dublin, Ireland; ^4^ZeClinics SL, Barcelona Biomedical Research Park, Barcelona, Spain

**Keywords:** visual cycle, inherited retinal degeneration, zebrafish, CRISPR/Cas9, CRALBP, RPE65

## Abstract

During the vertebrate visual cycle, all-*trans*-retinal is exported from photoreceptors to the adjacent RPE or Müller glia wherein 11-*cis*-retinal is regenerated. The 11-*cis* chromophore is returned to photoreceptors, forming light-sensitive visual pigments with opsin GPCRs. Dysfunction of this process perturbs phototransduction because functional visual pigment cannot be generated. Mutations in visual cycle genes can result in monogenic inherited forms of blindness. Though key enzymatic processes are well characterized, questions remain as to the physiological role of visual cycle proteins in different retinal cell types, functional domains of these proteins in retinoid biochemistry and *in vivo* pathogenesis of disease mutations. Significant progress is needed to develop effective and accessible treatments for inherited blindness arising from mutations in visual cycle genes. Here, we review opportunities to apply gene editing technology to two crucial visual cycle components, RPE65 and CRALBP. Expressed exclusively in the human RPE, RPE65 enzymatically converts retinyl esters into 11-*cis* retinal. CRALBP is an 11-*cis*-retinal binding protein expressed in human RPE and Muller glia. Loss-of-function mutations in either protein results in autosomal recessive forms of blindness. Modeling these human conditions using RPE65 or CRALBP murine knockout models have enhanced our understanding of their biochemical function, associated disease pathogenesis and development of therapeutics. However, rod-dominated murine retinae provide a challenge to assess cone function. The cone-rich zebrafish model is amenable to cost-effective maintenance of a variety of strains. Interestingly, gene duplication in zebrafish resulted in three Rpe65 and two Cralbp isoforms with differential temporal and spatial expression patterns. Functional investigations of zebrafish Rpe65 and Cralbp were restricted to gene knockdown with morpholino oligonucleotides. However, transient silencing, off-target effects and discrepancies between knockdown and knockout models, highlight a need for more comprehensive alternatives for functional genomics. CRISPR/Cas9 in zebrafish has emerged as a formidable technology enabling targeted gene knockout, knock-in, activation, or silencing to single base-pair resolution. Effective, targeted gene editing by CRISPR/Cas9 in zebrafish enables unprecedented opportunities to create genetic research models. This review will discuss existing knowledge gaps regarding RPE65 and CRALBP. We explore the benefits of CRISPR/Cas9 to establish innovative zebrafish models to enhance knowledge of the visual cycle.

## The visual cycle

**Figure 1:**
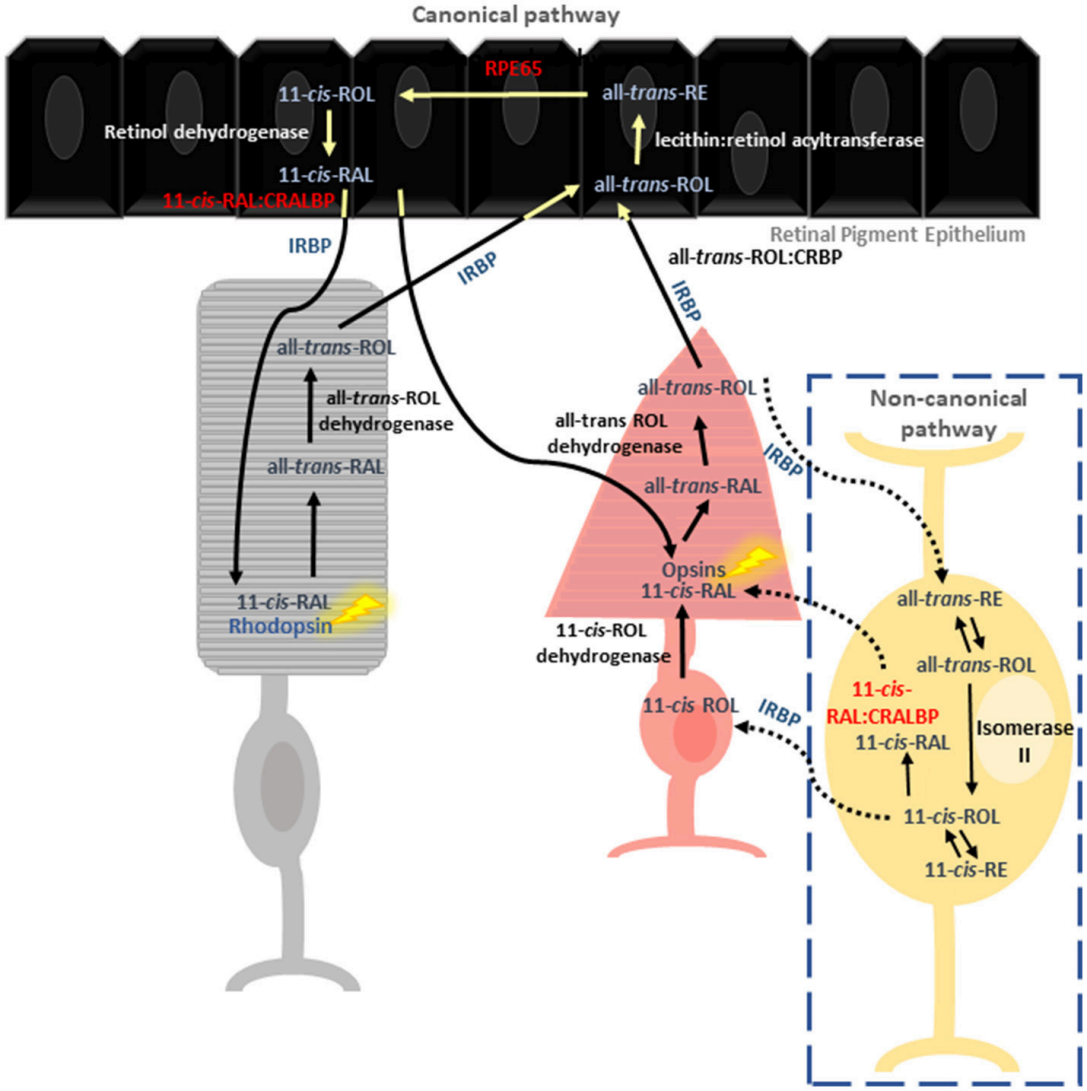
Reactions involved in chromophore regeneration in rod and cone photoreceptors. Rod pigment regeneration is limited to the canonical pathway where RPE65 isomerises 11-*cis-*RAL from RE. Isomerization occurs independently of RPE65 in the cone specific pathway, involving the Müller glia. CRALBP is thought to be the primary 11-*cis* retinoid carrier in both pathways.

The vertebrate visual system relies on two key pathways to detect and transform light signals into images. Phototransduction is vital for the conversion of external light stimuli, sensed by retinal photoreceptors, into electrical impulses that can be processed by the brain to form an image. Phototransduction is dependent on a constant supply of regenerated light-sensitive pigments. Thus, 11-*cis*-retinal (11-*cis*-RAL) is regenerated from all-*trans*-retinal (all-*trans* RAL) in a multistep enzymatic process known as the visual or retinoid cycle (Figure 1). The retinoid cycle is classified into the canonical and non-canonical pathways. Rod photoreceptors depend solely on the canonical visual cycle for regeneration of the photopigments, whereas, cones utilize both pathways (Kefalov, 2012).

### Canonical visual cycle in rod and cone photoreceptors

To date, most of our molecular understanding of the visual cycle is through the study of rod pigment regeneration. Rod photoreceptors rely exclusively on 11-*cis*-RAL supplied from the adjacent retinal pigment epithelium (RPE). Cones also use the canonical pathway, though to a lesser extent (Wang and Kefalov, 2011; Kiser et al., 2018). Absorption of photons by visual pigments within the photoreceptor outer segment triggers photoisomerization of 11-*cis*-RAL to all-*trans*-RAL, resulting in a conformational change in G-protein coupled opsin receptors and all-*trans*-RAL release. All-*trans*-RAL is then reduced to all-*trans*-retinol (all-*trans* ROL) by all-*trans*-ROL dehydrogenase, and shuttled to the RPE by interphotoreceptor retinoid-binding protein (IRBP) where it encounters cellular retinol binding protein (CRBP) (Kusakabe et al., 2009). In the RPE, all-*trans*-ROL is esterified by lecithin: retinol acyltransferase (LRAT) to produce retinyl esters (REs). Subsequently, the REs are isomerized into 11-*cis*-ROL by RPE65 and then oxidized into 11-*cis*-RAL by 11-*cis*-ROL dehydrogenase, prior to transportation back to the photoreceptor outer segments (Saari, 2016).

### Non-canonical visual cycle in cone photoreceptors

A second, cone-specific visual cycle pathway (also known as the intra-retinal visual cycle) is thought to have evolved through competition with rods for 11-*cis*-RAL or due to the greater chromophore demand of cones (Mata et al., 2002; Wang et al., 2009). In comparison to the well characterized canonical visual cycle, research in this area has lagged and as a result many questions regarding the function of key proteins and their role in disease pathologies remain unanswered. In this pathway, all-*trans*-ROL produced in the cone outer segment is processed in the Müller glia cells for regeneration of photoreceptor visual pigments. Within Müller glia, in the presence of cellular retinaldehyde binding protein (CRALBP) and a putative Isomerase II, all-*trans-*ROL is directly converted to 11-*cis*-ROL, which is subsequently transported to the cone inner segment by IRBP (Wang and Kefalov, 2011). Additionally, two novel enzymes identified within Müller glia–11-*cis* retinyl ester synthase and retinyl ester hydrolase were proposed to recycle 11-*cis*-ROL through an alternative means (Babino et al., 2015), however, their mechanism of action is not fully elucidated (Mata et al., 2002; Fleisch and Neuhauss, 2010; Saari, 2016).

This review focuses on the shortcomings in our understanding of the visual cycle, using two key proteins, RPE65 and CRALBP, as examples. Their pivotal role in human diseases as well as the emerging opportunities to develop zebrafish models harboring patient specific mutations using CRISPR/Cas9 genome editing technology is discussed.

### Spatio-temporal expression and functionality of RPE65 in the retina

RPE65, a 61 kDa retinoid isomerohydrolase, predominantly expressed in the RPE, is essential for regeneration of 11-*cis* ROL from all-*trans*-ROL (Redmond et al., 2005). Owing to its involvement in multiple human retinopathies and its central role in the visual cycle, this protein is extensively studied.

Interestingly, murine *Rpe65* expression is significantly downregulated in adult compared to fetal eyecups, providing evidence for developmentally regulated expression (Zhang et al., 2012). Teleost-specific whole genome duplication and divergence resulted in two *rpe65* orthologs in zebrafish. *rpe65a* and *rpe65b* have distinct spatial and temporal expression patterns (Schonthaler et al., 2007). At 4 days post fertilization (dpf), *rpe65a* is exclusively expressed in the RPE. In contrast, *rpe65b* is not detected in any light-sensitive structures during larval development and its expression is extinguished at 5 dpf. Knockdown of Rpe65a using morpholino oligonucleotides (MO) resulted in morphologically altered rod outer segments which suggested impaired visual function in *rpe65a* deficient larvae. Although 11-*cis*-RAL levels were reduced following dark adaptation, full regeneration occurred following dark re-adaptation. This study concluded that an Rpe65-independent isomerization event is necessary to sustain cone-mediated vision. However more recently, a third isoform, *rpe65c* was identified in zebrafish which is believed to be the result of a tandem duplication event. Similarly to *rpe65a*, it generates 11*-cis*-ROL from all-*trans* retinyl esters (Takahashi et al., 2011). Expression of *rpe65c* is localized to the inner retina, near the ganglion cell layer which equates with the location of Müller cell end feet. This is consistent with a possible role of Rpe65c in the non-canonical, intraretinal visual cycle.

Although its role in the alternate visual cycle is not fully characterized, *Rpe65* expression is reported in cones of multiple mammalian species (Znoiko et al., 2002; Wenzel et al., 2007), indicating an involvement in cone retinoid regeneration. Moreover “cone only” *Nrl/Rpe65*^−/−^ and *Rho/Rpe65*^−/−^ mice display reduced 11-*cis-*RAL levels and significantly decreased retinal sensitivity, thereby, connecting RPE65 with 11-*cis*-RAL production in the cone visual cycle (Wenzel et al., 2007). Disparately, others affirm the importance of Rpe65 in rod pigment regeneration but suggest cones rely on an alternative isomerase (Redmond et al., 1998; Wang and Kefalov, 2009). Evidence supporting a vitamin A isomerization event independent of RPE65 has emerged. Synthesis of 11-*cis-*ROL by RPE65 is much slower than the estimated rate of chromophore generation in daylight, suggesting the existence of an additional isomerization mechanism (Mata et al., 2002; Kaylor et al., 2013). In contrast to RPE65, which uses RE in the RPE as its substrate, previous studies documented 11-*cis*-ROL synthesis directly from all-*trans-*ROL in cultured Müller glia cells of the cone-dominant chicken (Das et al., 1992). Supporting an RPE65-independent isomerization event in cones, RE are less concentrated in the RPE of diurnal species in comparison to nocturnal ones (Mata et al., 2002). Dihydroceramide desaturase-1 (DES1), an enzyme expressed in Muller glia and RPE was revealed as a putative isomerase II. DES1 has a preference for the 9-cis chromophore however, it has also been shown to enhance 11-*cis*-ROL synthesis through interactions with RPE65 in the RPE (Kaylor et al., 2013). RNA-interference knockdown of *DES1* reduced vitamin A isomerization in Müller glia cells and *DES1* adenoviral gene therapy partially rescued visual impairment in *Rpe65*^−/−^ mice. Studies in salamander and mouse demonstrate that cones are supplied exclusively with 11-*cis* chromophore (Sato and Kefalov, 2016). More recently, cone dominant *Gnat1*^−/−^ mice treated with emixustat, a selective RPE65 inhibitor retained rapid recovery of cone light sensitivity following brief photobleaching, supporting the idea that 11-*cis* retinaldehyde production for this response relies on a RPE65-independent mechanism. Additionally, mice treated with emixustat exhibited slower resensitization following sustained photobleaching indicating a function of RPE65 in supporting cone function in prolonged light exposure (Kiser et al., 2018). Fenretinide, a DES1 inhibitor, decreased cone sensitivity in *Gnat1*^−/−^ mice, however, but did not completely abolish the intraretinal visual cycle to the extent of previous studies disrupting Müller cell function (Wang and Kefalov, 2009). Although promising, many visual cycle proteins (e.g., LRAT, retinol binding protein-4) are targeted by fenretinide, therefore, this response cannot be attributed to the reduction of DES1 alone. In summary, these data suggest context-specific roles for RPE-dependent and RPE-independent regeneration of 11-*cis*-RAL in cone photoreceptors.

### Spatio-temporal expression and functionality of CRALBP in the retina

CRALBP, encoded by *RLBP1*, is a cytosolic 36 kDa protein which binds *cis* retinoids with high affinity (Saari et al., 2009). In the mammalian retina, CRALBP expression is localized to the RPE and Müller glia cells and is involved in both rod and cone retinoid recycling (Bunt-Milam and Saari, 1983). Regeneration of visual pigments is delayed by absence of CRALBP (Saari et al., 2001) due to the inability to or inefficiency in associating and releasing retinoids from the ligand binding pocket (Golovleva et al., 2003). Although multiple roles of RPE-expressed CRALBP are elucidated e.g., binding of endogenous 11-*cis*-ROL/ RAL (Stecher et al., 1999), 11-*cis*-ROL dehydrogenase substrate carrier (Saari et al., 1994), and stimulation of RPE65 isomerization (Winston and Rando, 1998), the precise functionality of Müller cell-expressed CRALBP remains unknown. Co-immunoprecipitation experiments revealed that the Müller cell isomerase, DES1, interacts with CRALBP and synthesis of 11-*cis*-ROL is accelerated in the presence of CRALBP, suggesting a multiprotein complex involvement in cone retinoid recycling (Kaylor et al., 2013).

Moreover, *Rlbp1*^−/−^ mice display delayed dark adaptation with impaired cone function and 11-*cis* retinoid regeneration (Saari et al., 2001; Xue et al., 2015). In agreement, *Rlbp1*^−/−^ mice have impaired cone dark adaptation and loss of cone photoreceptors in addition to reduced ERG responses and M-opsin mislocalization (Xue et al., 2015). Additionally, the absence of functional CRALBP resulted in an accumulation of retinyl esters, demonstrating that isomerization is facilitated by the presence of functional CRALBP. Together, these results provide strong evidence that (1) CRALBP is essential for the maintenance of the intraretinal visual cycle and (2) the alternative pathway is crucial for cone mediated vision.

In zebrafish, two Cralbp isoforms (*rlbp1a* and *rlbp1b*) are present, with divergent expression patterns, allowing individual evaluation within the RPE and Müller glia, respectively (Collery et al., 2008). More specifically, *rlbp1a* is expressed in the RPE while *rlbp1b* is expressed in the Müller glia. Following knockdown of either isoform, a significant reduction in zebrafish optokinetic response (OKR) was reported (Collery et al., 2008). Others demonstrated a reduction in OKR and 11-*cis*-RAL production following knockdown of *rlbp1b* but not with *rlbp1a* (Fleisch et al., 2008). However, knockdown of *rlbp1a* or *rlbp1b* resulted in significantly reduced electroretinograms (ERG) supporting an independent requirement of both isoforms for cone vision (Fleisch et al., 2008). These discrepancies may reflect challenges in the reproducibility of transient MO knockdown experiments and illustrates the advantages of creating permanent knockout/knockin (KO/KI) models to further understand visual cycle biology.

## Diseases arising from mutations in genes associated with the visual cycle

The intricacy and complexity of the visual cycle is further evidenced by the multiple and diverse human retinopathies arising from inherited defects in this pathway. The biomedical significance of the visual cycle is irrefutable. However, the biochemical, molecular and cellular mechanisms of disease in these patients is often poorly understood. Retinal dystrophies associated with the visual cycle can be subdivided into two etiologies. The first is the impaired regeneration of light sensitive chromophore and the second involves the toxic accumulation of all-*trans*-retinaldehyde by-products.

Mutations in *RPE65* are causative of a large proportion (~11%) of early onset retinal degenerations (Thompson et al., 2000). As the sole isomerohydrolase in the RPE, defective RPE65 results in impaired all-*trans* to 11-*cis* isomerization and severely blinding disorders including autosomal recessive Leber congenital amaurosis (LCA) (Bainbridge et al., 2008), and dominant or recessive retinitis pigmentosa (RP) (Morimura et al., 1998; Bowne et al., 2011). The clinical manifestations arising from a range of amino acid substitutions represents the genetic and phenotypic heterogeneity of these conditions (Table 1). A diverse range of diseases arising from mutations in the same gene reflects the difficulty in clinically distinguishing RPE65 induced retinopathies. Consequently, diagnosis usually relies on progression and clinical presentation of disease. A diagnosis of juvenile RP is given to patients with photoreceptor degeneration who still retain some degree of central vision within the first decade of life (Booij et al., 2005). LCA is ascribed to patients with poor visual acuity together with nystagmus and an undetectable ERG before 1 year old (den Hollander et al., 2008). However, a previous study could not make this correlation (Morimura et al., 1998). The same study noted a child diagnosed with LCA, whose parents both had RP, exemplifying the genetic and clinical overlap of these conditions. An unanswered question requiring further study is to determine if the severity of disease correlates with the extent of residual function in mutated RPE65 isoforms.

**Table 1:** Pathologic mutations in human RPE65 and RLBP1 and the associated clinical symptoms.

**Gene**	**Disease**	**Onset**	**Mutation**	**Clinical manifestation**	**References**
RPE65	Leber Congenital Amaurosis (AR)	Early childhood	Leu67Arg^*^, Tyr368Cys, **Met1Thr**, **Gly40Ser**, **Arg91Trp, Glu102Lys, Asn321(1-bp ins), Val473Asp, Arg44Gln, Glu417Gln, Tyr144Asp, Trp350** + **3bp Ins, Tyr239Asp, Leu408Pro**	Cone-rod Dystrophy, Absent ERG, nystagmus, photophobia, amaurotic pupils, Nyctalopia	Morimura et al., 1998; Simovich et al., 2001; Philp et al., 2007; Xu et al., 2012
	Retinitis Pigmentosa (AR)	Juvenile/Young adult	**Arg91Trp**, Ala132Thr, **Leu341Ser, Glu404 – 4 bp Ins, Val452Gly, H68Y**	Rod-cone dystrophy; Night Blindness, Delayed dark adaptation, Bone-spicule deposits, attenuated blood vessels, optic disc pallor, visual field loss, and abnormal, diminished, or unrecordable ERG	Marlhens et al., 1998; Morimura et al., 1998; Samardzija et al., 2008
	Retinitis Pigmentosa (AD)	Juvenile/Young adult	**Asp477Gly**	Intra-retinal pigmentary deposits, chorioretinal atrophy, Reduced or absent ERG	Bowne et al., 2011
	Fundus Albipunctatus (AR)	Childhood	IVS1+5g → a^**^	Retinal thinning, multiple uniform retinal yellowish-white retinal lesions (subretinal flecks)	Schatz et al., 2011
RLBP1	Retinitis Pigmentosa (AR)	Juvenile/Young adult	**Arg150Gln**	Rod-cone dystrophy; Night Blindness, Delayed dark adaptation, Bone-spicule deposits, attenuated blood vessels, optic disc pallor, visual field loss, and abnormal, diminished, or unrecordable ERG	Maw et al., 1997
	Bothnia dystrophy (AR)	Childhood	**Arg234Trp, Arg233Trp**	Central scotoma, maculopathy, deposits with an appearance similar to bone spicules Retinal thinning, Reduced or undetectable ERG	Nojima et al., 2012
	Retinitis punctata albescens (AR)	Childhood	**(Gln278 - 1 bp del), Met226Lys, Arg 234Trp, Arg150Gln**, Arg233Trp; **Arg103Trp** + **Arg234Trp** (CH), IVS3+2 T → C^**^+ **Met225Lys** (CH)	Rod-cone dystrophy, Night Blindness, nyctalopia, white punctata throughout the fundus	Burstedt et al., 1999; Morimura et al., 1999; Gränse et al., 2004
	Newfoundland rod-cone dystrophy (AR)	Childhood	IVS3+2 T → C^**^; 324G → A	Night blindness from infancy; progressive loss of peripheral, central, and color vision, Reduced or absent ERG, attenuation of retinal vessels	Eichers et al., 2002
	Fundus albipunctatus (AR)	Childhood	**Arg150Gln; Gly116Arg**	Night Blindness, multiple uniform retinal yellowish-white retinal lesions (subretinal flecks), macular dystrophy	Naz et al., 2011

*Residue conserved in all zebrafish RPE65 orthologs **(bold)***.

* *Residue conserved in zebrafish rpe65c only*.

** *Intron sequence*.

CRALBP, encoded by *RLBP1* is a binding protein for 11-*cis* retinoids. *RLBP1* patient mutations are also linked to a wide array of autosomal recessive rod-cone retinopathies including RP, Bothnia dystrophy, retinitis punctata albescens (RPA), fundus albipunctatus (FA) and Newfoundland rod-cone dystrophy (Table 1; Morimura et al., 1999; Saari et al., 2001). Photoreceptor degeneration and night blindness are common features in these conditions; however, patients differ in disease onset and progression (Golovleva et al., 2003). Therefore, similar to RPE65, clinical diagnosis of *RLBP1* associated pathologies is commonly based on disease onset. A long-term study of an Arg150Gln pedigree revealed patients are diagnosed either with FA during their second decade of life, or with RPA during their fourth and fifth decades (Katsanis et al., 2001). The clinical distinctions between FA and RPA patients are subtle, leading to diagnosis based on clinical onset. Significant biochemical efforts assessed CRALBP ligand interactions (Crabb et al., 1998) and identified pathologic mutations (Liu et al., 2005). However, the molecular mechanisms that produce diverse genotype-phenotype disease correlations are poorly understood. Human blindness can also arise from defects in many other visual cycle associated genes. Mutations in the RDH12 also cause LCA, however, the clinical phenotype is not as severe as RPE65 dysfunction due to the involvement of multiple enzymes in all-*trans*-RAL conversion. Similarly, the RPE contains many enzymes capable of oxidizing 11-*cis*-ROL to 11-*cis*-RAL (e.g., RDH10 and RDH11); therefore, patients with *RDH5* mutations display a mildly progressive form of FA (Yamamoto et al., 1999). Stargardt disease is the most common form of juvenile maculopathy, usually with autosomal recessive inheritance. It can arise from mutations in the photoreceptor transmembrane rim protein encoded by *ABCA4*, resulting in impaired transport of vitamin A intermediates and accumulation of the toxic by-product A2E, a major component of lipofuscin. This results in secondary death of photoreceptors and a progressive loss of central vision. Additionally, *ABCA4* mutations are implicated in cone-rod dystrophy and recessive forms of RP (Cremers et al., 1998). Thus, the heterogeneous mutations in RPE65 and CRALBP are only a representative subset of the visual cycle genotypes linked to visual impairment or blindness. Genetic engineering of zebrafish models with germline knockout or patient-specific knock-ins of RPE65 or CRALBP mutations can provide a platform to study the underlying mechanisms leading to retinal degenerations. Potentially, these models can be exploited in chemical or genetic screens to identify novel therapeutic targets for patient-specific, precision medicine.

## Zebrafish as a model to study the visual cycle

A powerful approach to understand the etiology of visual cycle related disease is the creation and characterization of *in vivo* mutant models. As with most biomedical research, nocturnal rodent models dominate. Numerous knockout mouse models were developed to study visual cycle biology including: *Rpe65*^−/−^ (Redmond et al., 2005), *Rlbp1*^−/−^ (Saari et al., 2001), *Abca4*^−/−^(Charbel Issa et al., 2015), and *Rdh12*^−/−^ (Kurth et al., 2007). The *Rpe65*^−/−^ model is extensively utilized in pre-clinical gene replacement therapy experiments to restore rod and cone photoreceptor function. However, the discovery of a putative cone visual cycle necessitated development of cone dominant models. This was achieved using neural retina-specific leucine zipper (*Nrl*) knockouts that lack rod photoreceptors (Wenzel et al., 2007). Double knockouts with *Rpe65*^−^^/–^ allowed investigation of RPE65 functionality in the cone visual cycle (Wenzel et al., 2007). Although these models provide useful insights into the consequence of *Rpe65*^−/−^ and *Rlbp1*^−/−^ deletion, cones make up only 3% of the mouse retina and inevitably questions arise as to the relevance of rodents in studies relating to the cone-specific visual cycle.

Inherently, cone-dominant species including the chicken and ground squirrel support biochemical studies. In these models, enrichment of RE in the neural retina indicated a second pathway for cone pigment regeneration; as RE in rod-dominated retinae predominantly localize to the RPE (Berman et al., 1980; Mata et al., 2002). Indeed, the novel isomerase activity was later identified in a high-throughput expression screen of chicken retina (Kaylor et al., 2013). The cone-dominated chicken retina is powerful in the study of cone-specific proteins or cone function under healthy and disease conditions. Although some mutant lines are available, they are at risk due to the absence of a long-term housing facility (Delany, 2004). It is unlikely that the chicken model will replace the inexpensive rodent and fish models for routine genetic manipulation.

Owing to ~70% sequence identity between human and zebrafish orthologs, and ease of genome manipulation, zebrafish (*Danio Rerio*) are a powerful vertebrate model to study physiology and disease. Key advantages of zebrafish for vision research include rapid eye development and functional vision by 5 days post fertilization (Yin et al., 2012). Anatomical maturity of rods is not reached until 15 dpf and so, zebrafish larvae provide a valuable cone-dominated model of the visual cycle (Bilotta et al., 2001). At 5 dpf, vision is mediated by cone photoreceptors (Bilotta and Saszik, 2001; Chhetri et al., 2014). The optokinetic response (OKR), optomotor response (OMR) or electroretinography (ERG) can measure visual function in larvae at this stage (Daly et al., 2017). Similar to humans, cone-dominated, zebrafish retinae are thought to possess a second visual cycle pathway involving Müller glia and cone photoreceptors (Collery et al., 2008). The zebrafish genome is amenable to targeted editing and high efficiencies using CRISPR/Cas9 in this model have been reported (Liu et al., 2017). Other advantages of using zebrafish in visual cycle study include the ability to quantify retinoid levels in the eye, perform visual function assays on a large number of animals and to study cell specific functions of RPE65 and CRALBP due to gene duplication. Zebrafish reach sexual maturity at approximately 3 months old, therefore, a major limitation of their use in gene editing studies is the time required to establish an F_2_ generation to assess (~9 months). Additionally, complementary biochemical assays for CRALBP-retinoid binding or RPE65 isomerohydrolase activity must be performed *in vitro*.

### ENU mutant zebrafish lines available for Rpe65 and Cralbp

As highlighted above, key knowledge into the role of *rpe65* and *rlbp1* genes in visual cycle function and associated mechanisms of disease needs deeper investigation. From the zebrafish mutation project (www.sanger.ac.uk/science/collaboration/zebrafish-mutation-project), mutant alleles for all *rpe65* and *rlbp1* genes are recently available (Kettleborough et al., 2013) but characterization of these lines remains unreported. Two mutations in *rpe65a*, one at c.507>A and the second at c.1382C>T results in the introduction of a premature stop codon. Though both mutations are found within the functional domain of RPE65, the second mutation occurs close to the C′ terminus, thus, a fully functional Rpe65a protein may be retained. For *rpe65b*, three mutant alleles c.238T>A, c.433T>A, and c.1032T>A are available. All three are expected to result in nonsense mutations and truncated Rpe65 proteins. For *rpe65c*, c.251T>C, is predicted to affect an essential splice site. For *rlbp1a* and *rlbp1b*, a single point mutation in each has been identified, c.209A>T and c.234T>G respectively, that are deemed to be nonsense mutations. In both cases the mutations are closer to the N' terminus and the mutant protein is predicted to be truncated and non-functional due to protein mis-folding and/or targeted for early degradation.

These forward genetic mutants harbor single point mutations with random localization. Hence, they may not provide the most suitable model to understand *rpe65* or *rlbp1* function or dysfunction (Rossi et al., 2015). Additionally, compensation may occur in zebrafish if the mutation consists of short deleted fragments or if the mutation occurs close to the N′ terminus (Prykhozhij et al., 2017). Therefore, CRISPR/Cas9 is ideal for generating targeted mutations to address specific questions.

## CRISPR/Cas9 genome editing

At the end of the twentieth century, publication of the results from several large-scale forward genetic screens in zebrafish represented a major breakthrough in vertebrate genetics (Nüsslein-Volhard, 2012). Use of N-ethylnitrosourea (ENU), an alkylating agent which causes random point mutations created over 4,000 isolated mutants, 400 of which displayed a visible phenotype and importantly, had a human ortholog. This demonstrated the relevance of zebrafish. The mutations typically resulted in hypomorphic or null alleles. Notably, many human genes have more than one zebrafish ortholog, which is beneficial when modeling lethal phenotypes and tissue specific functions. However, a common problem often associated with the presence of multiple isoforms is redundancy or compensatory functionality. Targeted genome editing has become a powerful approach to study gene function. Initial approaches relied on zinc finger nucleases (ZFN) and transcription activator-like effector nucleases (TALEN) which were technically challenging and associated with off-target effects. Notably, TALEN technology was successfully applied to generate zebrafish models to study retinoid metabolism (Shi et al., 2017) and the role of ciliary genes in cone photoreceptor development and survival (Lessieur et al., 2017). However, CRISPR/Cas9 gene editing is now the preferred option. Here, we discuss the opportunities to investigate visual cycle biology by creating patient-specific KO and KI models in zebrafish.

### CRISPR/Cas9 as a genome editing tool

The advances in CRISPR/Cas9 technology represents a new generation of targeted genome editing which allows researchers to specifically control gene function. CRISPR/Cas9 has been adapted from a type II bacterial defense system which enables host degradation of exogenous sequences from invading bacteriophages. CRISPR/Cas9 is now exploited for the generation of many modified organisms and cell lines (Munoz et al., 2014; Yang et al., 2014; Albadri et al., 2017; Champer et al., 2017). The specific endonuclease activity of CRISPR/Cas9 relies on three components: Cas9, a RNA-guided endonuclease from the type II CRISPR system, a trans-activating RNA (tracrRNA) and a CRISPR RNA (crRNA). Both RNAs can be fused to produce a chimeric single guide RNA (sgRNA) targeting an 18–25 base pair (bp) sequence. Algorithms for designing sgRNAs and predicting on and off target effects are open access (Gagnon et al., 2014; Hwang et al., 2015; Moreno-Mateos et al., 2015). Multiple sgRNAs can be co-injected making CRISPR/Cas9 extremely useful in creating zebrafish knockouts, as all orthologs can be knocked out simultaneously to overcome gene compensation. The primary restriction of sgRNA design is that a protospacer adjacent motif (PAM) recognized by Cas9 sequence must be adjacent to the 3′ end of the target sequence. To overcome this issue, Cas9 variants are being created which recognize a broad range of PAM sequences (Hu et al., 2018). The widely used *Streptococcus pyogenes* derived Cas9 recognizes a PAM sequence of 5′-NGG. When co-injected with an appropriate sgRNA, a blunt double stranded break (DSB) is introduced three nucleotides upstream of the PAM. Upon DSB, the native host DNA repair mechanism known as non-homologous end joining (NHEJ) promotes insertions or deletions (indels). These indels result, in most cases, frameshifts and incorporation of early stop codons, creating gene KOs, enabling functional genomic studies (Liu et al., 2017). NHEJ can generate reporter lines but are not suited for precise genome editing (Auer et al., 2014; Kimura et al., 2014). Alternatively, a donor template flanked by homologous end sequences can be co-injected to be integrated at specific mutations/regions in the endogenous gene through homology directed repair (HDR) (Jasin and Haber, 2016). Incorporation of point mutations through HDR is difficult but success has been reported (Irion et al., 2014; Armstrong et al., 2016). An advantage of zebrafish for gene editing is that efficiencies can be determined within 2 days and stable germline transmission be verified in F_1_ offspring ~3 months later. Several aspects, such as the conformation and purity of the donor DNA and the length of the homologous flanking sequences affect HDR efficiency (Hisano et al., 2015; Hoshijima et al., 2016; Zhang et al., 2016). Moreover, prior sequencing of relevant genomic regions can aid in avoiding regions of methylated and inaccessible chromatin (Sertori et al., 2016).

More recently, CRISPR capability has extended to gene silencing (Kolli et al., 2017), transcriptional activation (Chavez et al., 2015), gene labeling (Khan et al., 2017), and conditional knockouts (Miura et al., 2017). Mutations in the two nuclease domains, HNH and RuvC, render Cas9 catalytically impaired (deactivated or dCas9) which enables repurposing of the well-established CRISPR/Cas9 system for specifically targeting genomic DNA without cleaving it (Qi et al., 2013; La Russa and Qi, 2015). Through blocking of RNA polymerase activity upstream of the sgRNA, Qi et al. demonstrated the use of dCas9 in gene repression in *E. coli* (Qi et al., 2013). Similar to RNAi, this method, known as CRISPRi is reversible via use of an inducible promoter to control dCas9 expression. Despite concerted efforts applying RNAi in zebrafish, widespread off-target effects were problematic (Wargelius et al., 1999; Gruber et al., 2005). CRISPR-mediated gene activation (CRISPRa) utilizes fusion proteins to recruit transcription activators (La Russa and Qi, 2015). Correcting a pathogenic mutation through single base editing remains an attractive possibility of CRISPR/Cas9. Editing single bases through HDR is largely associated with incorporation of stochastic indels as a result of competing NHEJ mechanisms (Rees et al., 2017). Although challenging and inefficient, some groups have been successful in editing single base pairs in zebrafish (Armstrong et al., 2016; Rees et al., 2017; Zhang et al., 2017). Additionally, to overcome issues with off-target effects, single base pair editing, without inducing double stranded breaks is now feasible using a modified Cas9 consisting of Apolipoprotein B MRNA Editing Enzyme Catalytic Subunit 1 (APOBEC1) enzyme and a uracil glycosylase inhibitor fused to nickase Cas9 (Armstrong et al., 2016; Kuscu et al., 2017; Zhang et al., 2017). Novel single nucleotide polymorphism (SNP) mutations are constantly being uncovered in RPE65, in particular, however as many SNP changes can be randomly found in one gene, it is unknown which are pathogenic, if any (Mo et al., 2014). The development of routine single base pair editing has opened up opportunity to model emerging SNP changes in zebrafish to study the effect of these mutations.

### Strategies to target *rpe65* and *rlbp1* structural and functional domains using CRISPR/Cas9

To date, ~120 mutations have been identified in *RPE65*, resulting in heterogeneity in severity and disease pathogenesis (Jin et al., 2016). It is vital that future studies target human variants of *rpe65* to further understand the link between the mutation, RPE65 functionality and disease pathogenesis. Generating zebrafish models with the most common human mutations can elucidate the mechanisms leading to vision loss in patients.

Using CRISPR/Cas9, we can selectively target key amino acids critical for RPE65 protein folding and structural stability, active sites and co-factor binding sites. Some patients harboring mutations in RPE65 retain residual vision, therefore it would be valuable to understand the underlying mechanisms which preserve functioning vision. Moreover, the consequences of amino acid substitution are normally predicted based on their location; therefore, selectively targeting these residues in zebrafish would provide an *in vivo* platform to test these predictions. The Arg91Trp is a common disease-causing alteration in the RPE65 gene. Studies *in vitro* and *in vivo* highlighted reduced RPE65 stability and isomerase activity; however, the mouse retina possessed residual isomerase activity unlike the cell line expressing the variant. Zebrafish could be used as a powerful tool in resolving discrepancies between *in vitro* and rod-dominant rodent studies. In zebrafish, *rpe65c* includes four conserved histidine residues for iron binding and a palmitoylated cysteine residue for membrane association and it has been suggested that rpe65c-mediated conversion of 11-*cis*-ROL depends on iron availability (Takahashi et al., 2011). Therefore, specifically targeting the *rpe65c* iron binding domain can test the hypothesis that isomerase activity will be impaired. Additionally, crystal structures predict RPE65 functions as a homodimer (Kiser et al., 2012). Targeting sequences involved in Rpe65 dimerization would affect protein folding and function and test this theory. Consideration should be given to creating zebrafish lines with mutations in the catalytic domain of RPE65, resulting in a constitutively active protein.

Similar considerations should be applied for designing *rlbp1a* and *rlbp1b* CRISPR/Cas9 KO or KI models. Functional characterization of mutant lines will improve understanding of the requirement of *rlbp1a* and *rlbp1b* in RPE and Müller glia cells, respectively, as well as in the mechanism of retinal diseases. It will be fascinating to alter the sequences reported to be involved in maintenance of the structural integrity of the ligand binding pocket and assess effects on retinal histology and vision *in vivo* (He et al., 2009). Additionally, targeting specific amino acid residues in CRALBP could aid in validating previous *in vitro* binding studies. For example, recombinant CRALBP with the R150Q or M225K substitution were found to be less soluble than WT CRALBP and lack the ability to bind 11-*cis-*retinaldehyde.

Investigation into loss or gain of function mutations in specific *rlbp1* and *rpe65* isoforms is particularly appealing. It has been suggested that CRALBP interacts with retinoid isomerases in the RPE and Müller glia cells (Kaylor et al., 2013). By studying individual isoforms, one can understand the roles of these two cell specific isoforms independently; and in combination, to obtain a complete picture of the functionality of these two key proteins within the visual cycle.

### Understanding RPE65 and CRALBP in disease pathogenesis using novel zebrafish CRISPR/Cas9 lines

We are still in the initial phase of exploring the possibilities that CRISPR technology offers for gene regulation and the control of cell identity and behavior. At present, gene activation, gene silencing, KI and KO are all feasible in zebrafish and their use in retinal regeneration studies has recently been reviewed (Campbell and Hyde, 2017). The most powerful and straightforward strategy to probe gene function is to characterize the consequence of its deletion. The creation of *rpe65*^−/−^ and *rlbp1*^−/−^ zebrafish lines using CRISPR/Cas9 technology provides an exciting opportunity to study visual cycle processes and dissect the molecular mechanisms leading to RPE or photoreceptor loss in patients. The potential benefits associated with the generation of zebrafish through CRISPR/Cas9 editing are: (1) to improve our understanding of the biochemical, molecular and cellular progression of diseases associated with the visual cycle and (2) to develop novel gene, cell and drug based therapies for human pathologies linked to defective RPE65 or CRALBP.

In humans, high-acuity vision is predominantly mediated by cones. It is therefore imperative to understand the pathophysiology of the *RPE65*^−/−^ and *RLBP1*^−/−^ phenotype specifically in relation to cone photoreceptors. Unanswered questions regarding the visual cycle mainly relate to the cone specific visual cycle. In line with previous studies supporting the existence of a putative cone specific visual cycle (Das et al., 1992; Mata et al., 2002; Kaylor et al., 2013), it will be interesting to determine if knocking out critical visual cycle genes in the zebrafish retina also supports this idea. While there is much evidence supporting the existence of this pathway (Das et al., 1992; Mata et al., 2002; Wang and Kefalov, 2011), there is a lack of functional evidence leading researchers to question the physiological relevance of these studies. Knockout of Rpe65 and Cralbp function in cone-dominated zebrafish will provide complementary data to these studies in addition to previous MO-mediated knockdown studies conducted in zebrafish (Schonthaler et al., 2007; Collery et al., 2008). For example, it has been argued that *trans-cis* isomerization in zebrafish is an Rpe65 independent process (Schonthaler et al., 2007) however, at this point in time, Rpe65c had not been identified (Takahashi et al., 2011). Knocking out individual isoforms as well as creating double and triple zebrafish knockouts will determine if Rpe65 is required in the cone-specific visual cycle.

The rapid advances in CRISPR/Cas9 technology may mean that virtually any mutation can be created *in vivo* and extends its potential use to personalized zebrafish knockouts for specific patient mutations. For example, patients harboring a R91W RPE65 mutation retain low levels of 11-*cis* chromophore generation and some cone function during early life (Samardzija et al., 2008). A *Rpe65* hypomorphic murine model has been generated for this specific mutation and crossed with *Nrl*^−/−^ to study cone function (Samardzija et al., 2016) and similarly to patients, the degenerative phenotype is mild but progressive (Hull et al., 2016). More recently, a Asp477Gly change has been identified in a pedigree with similar pathology; however this is yet to be mimicked *in vivo* (Bowne et al., 2011). It is postulated that the introduced mutation consequently causes the destabilization of protein folding, though the exact mechanism is not understood or well-studied. With these findings, it raises the possibility that more patients with gain of function mutations may be discovered in coming years. Logistical and technical difficulty remain as barriers in creating knockout rodent models; therefore, it is unlikely that mouse lines with rare human mutations will be made. Creation of custom-made zebrafish models harboring these mutations will enhance our understanding of the pathophysiology arising from such novel mutations.

Interestingly, some vision loss patients retain residual RPE65 activity with reduced and partial vision, as a result. An alternative approach to study human variations in RPE65 is to constitutively activate the gene of interest through KI of the human specific mutation. This will be extremely valuable as the effect of retaining some RPE65 isomerization activity on photoreceptor integrity and function can be studied. Similarly, an arginine to glutamine substitution in CRALBP at position 150 results in autosomal dominant RP. *In vitro* studies show this mutation abolishes 11-*cis* retinoid binding and reduces CRALBP solubility (Maw et al., 1997). CRISPR mediated KI of the Arg150Glu mutation in zebrafish could identify additional functions of CRALBP *in vivo* and the processes leading to CRALBP mediated RP degeneration.

Gene replacement therapies for RPE65 have recently received FDA approval and ones for recessive CRALBP mutations are in development (MacLachlan et al., 2018). However, these are expensive and are not widely accessible to patients. The molecular basis leading to RPE or photoreceptor loss in patients remain poorly understood. As a result, there are no approved drug therapies successful in slowing or preventing visual cycle related retinal degeneration. The use of a 9-*cis*-RAL analog is being evaluated in LCA patients following its beneficial use in restoring light sensitivity in *Rpe65*^−/−^ albeit concerns remain over the adverse drug responses reported (Maeda et al., 2013; Scholl et al., 2015). Conversely, CRISPR/Cas9 gene editing is being explored as a gene therapy in many conditions, including retinopathies (Huang et al., 2017; Smith et al., 2017). Mutations in *RPE65* and *RLBP1* typically lead to slow progressing rod-cone dystrophies and therefore, the application of effective therapies during early disease presentation could preserve remaining photoreceptor structural integrity and retain functional vision. Zebrafish are useful in identifying lead compounds before application in a mammalian model and developing *rlbp1*^−/−^ and *rpe65*^−/−^ lines would provide an inexpensive platform to conduct high throughput pharmacological screens.

## Concluding remarks

Although our knowledge of visual cycle processes and related human pathologies has progressed significantly in the last decade, our understanding of the cone-specific visual cycle and the biochemical mechanisms leading to photoreceptor degeneration is lacking. To advance therapeutics for degeneration, caused by defective visual cycle genes, it is necessary to develop robust and reliable *in vivo* models. Targeted gene editing using CRISPR/Cas9 KO and KI strategies are routinely applied in zebrafish with very high success rates. Development of a permanent KO or KI cone dominant models will provide inexpensive and reliable platform to study the resultant phenotypes in the long term and advance our understanding of the molecular processes which lead to cone degeneration in patients.

## Author contributions

RW was the primary author of this review. HS and VD contributed in drafting sections for this review. HS prepared the figure. BK contributed significant intellectual input and approved the submission of the review. All authors edited the final draft.

### Conflict of interest statement

VD is employed by ZeClinics SL. The other authors declare that the research was conducted in the absence of any commercial or financial relationships that could be construed as a potential conflict of interest.

## Glossary

A2E: – N-retinylidene-N-retinylethanolamine
All-*trans*-RAL: – All-*trans*-retinal
All-*trans*-ROL: – All-*trans*-retinol
bp: – Base pair
CRALBP: – Cellular retinaldehyde binding protein
CRBP: – Cellular Retinol binding protein
CRISPR: – Clustered Regulatory Interspaced Short Palindromic Repeats
CRISPRa: – CRISPR activation
CRISPRI: – CRISPR interference
dCas9: – Deactivated Cas9
DES1: – dihydroceramide desaturase-1
dpf: – Days post fertilisation
DSB: – Double Stand Breaks
ENU: – N-ethyl-N-nitrosourea
ERG: – Electroretinography
FA: – Fundus albipunctatus
GPCR: – G protein coupled receptors
HDR: – Homology directed repair
Indels: – insertions or deletions
KI: – Knockin
KO: – Knockout
LCA: – Leber Congenital Amaurosis
LRAT: – Lecithin Retinol Acyltransferase
MO: – Morphilino Oligonucleotide
NHEJ: – Non- Homology end joining
Nrl: – neural retina-specific leucine zipper
OKR: – Optokinetic Response
OMR: – Optomotor Response
PCR: – Polymerase Chain Reaction
RE: – Retinyl Esters
RP: – Retinitis Pigmentosa
RPA: – retinitis punctata albescens
RPE: – Retinal Pigment Epithelium
SNP: – Single Nucleotide Polymorphism
TALEN: – Transcription activator-like effector nucleases
WT: – Wildtype
ZFN: – Zinc Finger Nucleases
11-*cis*-RAL: – 11-*cis* retinal
11-*cis*-ROL: – 11-*cis* retinol
